# Oxygen, Iron and the Spinal Ice Bag in the Treatment of Anemia

**Published:** 1897-09

**Authors:** Beverley O. Kinnear

**Affiliations:** New York


### OXYGEN, IRON AND THE SPINAL ICE BAG IN THE TREATMENT OF ANEMIA.

BY BEVERLEY O. KINNEAR, New York.

THE second of these remedies is considered, doubtless, by the majority of the profession as the most efficient medication in anemia, and as a single agent it appears to be the one which has been more universally given than any other and from which the best results have been obtained.

It is the desire of the writer—in a concise paper—to lay before the profession some reasons which denote to him, not without much cogency, why the combination of the three above-mentioned remedies should meet the indications of this disease better than iron alone, or oxygen, or the spinal ice bag alone or in combination. The three prominent factors in anemia are : decrease in the number of the red corpuscles of the blood ; a lack of normal warmth in the extremities, demonstrated by the chronically cold hands and feet ; and a feeble pulse, yet constant excitability of the nervous system. The first is an indication for the use of iron, the second denotes the craving of the tissues for a larger supply of oxygen and the third implies systemic weakness and excitement of the brain and nerve centers. The sympathetic ganglia are evidently functioning with undue force, as shown by the cold surface of the body.

If we can, at the same time, increase the number of the red corpuscles, dilate the systemic arteries and thoroughly oxidise the blood and tissues, will we not be adding effective force to force and thus much more rapidly ameliorate the conditions and cure the disease, than if we either relied upon one of these remedies or used only one at a time ? There is nothing incompatible in their conjunction. Oxygen by the lungs, iron by the stomach and the mechanical action of cold over the spine upon hyperemic nerve centers—the first stimulating the cells of all tissues to renewed action and aiding in the assimilation of the food ; the second rapidly increasing the number of the red corpuscles and restoring the hemoglobin to the healthy standard and the third dilating the systemic arteries, so that a much larger supply of the reoxygenated blood is carried to every organ, muscle and tissue in the whole body. It appears to us that such a treatment for anemia is about as perfect, with our present light, as treatment can be.

In acute anemia, after exhausting hemorrhages, we would not advise the use of the spinal ice bag, except in the hands of an expert, but rather the inhalation of oxygen alone to restore the vital powers, quiet the pulse and improve the nutrition. After all danger of hemorrhage has passed, then the spinal ice bag of twenty-two inches in length, and placed over the spine from the fourth cervical to the third lumbar vertebra, may be added with much benefit, as well as the internal administration of iron.

In acute anemia we have cold sweats, great pallor, rapid, feeble pulse, easily excited by mental or bodily exertion to greater speed, pinched features and a blowing cardiac murmur. There are prominent nervous symptoms, delirium, tinnitus aurium and the like, and there is intense thirst.

Cases of this kind can be more speedily relieved by the inhalation of oxygen than by iron or by the spinal ice bag, but may be followed, as the patient improves, by the use of both, in order to hasten and perfect the cure.

Dr. John V. Shoemaker, in his treatise on materia medica and therapeutics, says of oxygen :

It first quickens, then slows the pulse, but makes it fuller and rounder. In addition, there is evidence in the lips and finger nails of increased oxygenation of the blood, and cicatrising wounds and granulation tissues were observed by Demarquay to become more ruddy. The digestion and appetite improve, with increased assimilation and resulting physical strength. Expiration of carbon dioxide is largely increased. In chronic cachectic states it improves assimilation by building up of the system.

The description here given demonstrates that metabolic changes become much more rapid during oxygen inhalation, and as an after-effect also; therefore the processes of repair and waste are soon brought to the normal.

The very same results as above given we have obtained in many chronic anemic cases by the application of the spinal ice bag alone, illustrating thereby the similarity of action of oxygen by inhalation and the cold applications. Cold over the spine dilates the arterioles throughout the body, generates heat within the body at the same time and deepens respiration, therefore supplies more air, and therefore more oxygen to all the tissues of the body, but these results are gradual and can be much hastened by the use of iron and oxygen at the same time.

. In the discussion following the reading of a paper at the Academy of Medicine, New York, by Professor W. Gilman Thompson, in May, 1889, Dr. J. West Roosevelt stated "that in the simple anemia of girls he found the patients do better under the use of oxygen in connection with the administration of iron than under the iron alone." This statement was confirmed at the same meeting by Dr. Beverley Robinson, of New York. The action of iron alone appears to be not only that of a red corpuscle generator, but to act somewhat similarly to the ice bag, and to the inhalation of oxygen by increasing tissue oxidation, toning the heart, improving the nutrition by its effect upon the circulation, and it is supposed to convert oxygen in the blood into ozone, thus promoting oxidation in muscles, organs and tissues. Thus it may be very easily seen why these three remedies used together will each intensify as well as supplement the action of the others and thus the more speedily ameliorate the suffering and cure the disease. Upon the administration of iron, that is the particular preparation to be used, we have little to say, beyond the caution : do not use large doses, for very little iron is absorbed and there is much less fear of upsetting the stomach than if large doses be given, especially when the more astringent preparations are prescribed. Upon oxygen and the application of the spinal ice bag we must be much more explicit, as they are little understood by the majority of the medical profession.

Dr. Hayem, professor of therapeutics and materia medica in the medical faculty of Paris, France, says : "The immediate action of oxygen is doubtless to render the action of the heart and the arteries stronger, the remoter effects being an increase in the red color of the blood when pale, and a heightened complexion." To the writer this testimony evidences three very interesting and important points.

The first is the immediate action of the oxygen upon the heart and the arteries, or in other words, its action at once upon the active muscular fiber, or the muscle cell in a state of activity. The arterial system including the heart, or the involuntary muscular apparatus, is always at work independently of our will and the first effect of oxygen, according to this high authority, is upon the involuntary yet constantly active muscular fiber. Thus the immediate action is to strengthen the action of the heart muscle, increasing its contractile power and increasing the nutrition of the organ.

The second point, and it seems to us equally instructive, is the stronger action imparted to the arterial system. This cannot be alone due to the toning of the heart muscle, for we know that there is a *vis a fronte* exerted upon the capillary system, as well as the *vis a tergo*, or the impulsion given to the circulation of the blood by the heart muscle.

What conclusion may we draw, then, from this stronger action of the arterial system ? There are two deductions. The first is that the inhaled oxygen being quickly distributed to every organ and tissue in the body, directly stimulates the cells of all tissues to more vital effort, causing a fuller flow of blood through the capillaries, and therefore more natural chemical action between the tissues and the blood, and so an increase of heat and of the reparative processes.

The second is that the oxygen stimulates at once the cerebro-spinal vaso-dilator centers, causing them to function more actively and immediately dilate the arterioles throughout the body. Through physiological experiment we know that when the cerebro-spinal centers or nerves are stimulated, when terminating in the glandular system, the arteries in the glands dilate and there is an increase of discharge from the gland. But Brown-Sequard also demonstrated an increase in the activity of the circulation in the muscle and tissues of the tongue by stimulation of a cerebro-spinal nerve, therefore a dilatation of the arterioles in that organ; and this fact tends to prove that the *vis a fronte* of the capillaries is derived from the central nervous system. We have long believed this latter statement to be true, because by the use of heat over the spine in bronchial inflammations, for example, not only does there result a contraction of the dilated arterioles in the bronchial mucous membrane, but with this relief, there is an immediately responsive action of the mucous glands in the bronchial area, and a profuse and easily expectorated discharge. We have had this result in a large number of patients, the heat stimulating the function of the dorsal sympathetic ganglia (over which it is placed in bronchitis) and thus inducing contraction of blood-vessels in the inflamed mucous membrane, and at the same time stimulating those spinal cells which issue nerves to the nuclei of the mucous membrane cells of the bronchi, resulting in increased discharge from the glands. At least this has been the only explanation which has seemed to us sound; and it is corroborated by physiological experiment, microscopic anatomical research connected with nerve terminals, and the clinical observation of other educated physicians.

The third point of Dr. Hayem's testimony is that the "red color of the blood when pale is increased," and this, it seems to us, can only mean an increase in the red corpuscles and, therefore, a similar result to that following the administration of iron. The inhalation of oxygen, then, is not only indicated in all cases of anemia, but in heart failure and heart disease, whether functional or organic, and also in all cachectic and feeble conditions.

In an interesting paper by Dr. W. T. Baird, read before the Mississippi Valley Medical Association, entitled Oxygen as a heart tonic, he says (1) oxygen is essentially a muscle food; (2) the *active* muscle cell consumes the food. And the conclusion drawn is that the heart first receives the stimulus of oxygen when inhaled.

We cite from his article a most interesting case of anemia treated by oxygen inhalation:

Mrs. B., age 43, multipara, has been a confirmed invalid for two years; is very pale and anemic, suffering from a complication of diseases apparently induced by the unilateral laceration of the cervix uteri, (the cicatrix of which is filled with indurated cicatricial tissue), resulting in various reflex neuroses and profound anemia, with weakness and dilatation of the heart walls. Debility extreme; unable to be up but a few hours each day. Suffers from insomnia and loss of appetite to the extent that food has to be forced upon her. Constant melancholy and intense pressure on the brain; takes no interest in family, and has sexual orgasms many times day and night. Anemic murmurs, plainly heard with first sound of the heart over apex. Pulse 140, weak and irregular. Enough flesh, but it is soft and flabby. I concluded to administer ten gallons of oxygen daily. Commenced with the administration of that amount by inhalation on the 6th day of October last, and in the course of a few days her husband reported that she was resting better at night, rising earlier in the morning, and exhibiting more strength; calling for food for the first time in a year and, in short, manifesting a decided improvement in all her symptoms.

Her heart now (November 17, 1894,) shows but little dilatation, her pulse 120, fuller and softer, and her complexion ruddy. Her sexual orgasms have entirely ceased. She is more cheerful, complains less of her head, and upon examination of the cervix I find cicatricial induration decidedly less, and this accounts for the amelioration in her reflex symptoms, as the peripheral nerves in the cervix are thereby partially set at liberty. This can only be accounted for by an improved circulation, as a result of the improvement in tone in the muscular fibers of her heart, followed by a general toning of the whole system, and the renewal of functional activity in all of its organs.

Our comment upon this case is, that the improvement in the circulation in the womb was probably due to the *vis a fronte* action of the oxygen upon the capillary circulation.

Oxygen, as a therapeutical agent, is but little understood by the medical profession at large. It is a mistake to suppose that ordinary commercial oxygen, such as every calcium light maker manufactures, is a proper agent to be used for therapeutical purposes. It is saturated with chlorine and other deleterious gases, which are injurious to the patient, as they are exceedingly poisonous. Therefore in the employment of oxygen it is necessary to obtain the very best quality. Also we would call attention to the fact that oxygen should never be used alone, even when pure, as it is too powerful an oxidising agent, frequently exciting irritation, especially in those cases where there is subacute inflammation. It must, therefore, be diluted with an agent of lighter specific gravity than oxygen, and we find nitrous monoxide to be the most valuable for this purpose.

The formula which seems to us to be most suitable in cases of anemia is composed of two parts of pure oxygen, one part of nitrous monoxide, and we consider the addition of a small equivalent of ozone valuable, as it keeps the oxygen fresh. We are in the habit of obtaining oxygen fresh from Dr. Walton's laboratory in New York, where this formula may be obtained, or any other that in the judgment of the physician seems best suited to the case. We find no objection to the use of oxygen compressed in cylinders, provided a small quantity of ozone is used to prevent the gas from rapid deterioration.

The formula referred to is now being used by the London Oxygen Hospital, and we are under the impression that Walton's gas can be obtained almost anywhere from all prominent druggists, although we prefer to obtain the gas fresh from the laboratory in New York. We speak with much emphasis in regard to the formula and the quality of the gas, as both are exceedingly important in order to obtain satisfactory results.

Some physicians are reluctant to employ oxygen, as they hesitate to order it compressed in steel cylinders for fear that the mechanism might not be easily understood. The gas is drawn from the cylinder just as we draw water from a water faucet—there is a valve which, upon being turned, allows the gas to escape. A child can easily manage it, therefore there is no reason why this most valuable agent should be neglected because of ignorance in regard to its manipulation.

In severe cases of acute anemia, oxygen may be administered constantly until relief is obtained, and then repeated as often as the exigencies of the case require ; three times a day as the case improves. Close the nostrils, place the tube in the mouth after complete expiration and then inhale to the fullest capacity of the lungs ; hold as long as is comfortable and exhale slowly through the nasal passages. Wait two minutes and repeat the process. Do this three times a day in ordinary anemic cases, whether acute or chronic, and have the patient stand, if possible, during inhalation of the gas.

The spinal ice bag (twenty-two inches in length, four and a quarter in breadth,) may be used in all cases of chronic anemia with benefit, except in those cases where there is very evident and troublesome hyperemia of the head. It can be applied, first wrapped in one thickness of flannel, over the spine from the fourth cervical vertebra downward, being placed so that the bag lies equally upon each side of the vertebral spines. It may be used for one half an hour three times a day, morning, noon and evening, one hour after meals. If the hyperemia of the head is a very prominent symptom, then only fill the upper two compartments of the bag and apply over the spine from the fourth dorsal vertebra downward. This method of application will dilate the arterioles in the lower two-thirds of the body, and thus assist in relieving the hyperemia of the head.

One illustrative case treated and cured by the use of the spinal ice bag some years ago, when in practice in Boston, Mass., will serve to show the results which may be secured by an intelligent use of heat and cold over the spine in anemia; and I speak of heat as well as cold because the hot water bag will often be of service in relieving local congestions, such as hyperemic headache and that tendency to uterine hemorrhage which we often find in married women suffering from general anemia.

Mrs. F., thirty years old, had been confined to the house, and most of the time to her bed, for nineteen months previous to her treatment by the spinal ice bag, and had tried a host of remedies without success. She suffered with constant headache, was exceedingly nervous, had almost no appetite, could not digest and assimilate well what nourishment she did take, slept little and body, arms and legs were exceedingly cold all the time, evidencing a powerful contraction of the capillaries, and therefore feeble nutrition of the muscular system in general. Her bowels were constipated and she was much troubled with mental depression, as well as with great physical prostration. She was so weak that she could not walk across her bedroom. The spinal ice bag was used, a full length bag, for forty minutes each night and morning, and she at once began to improve in every way. Her sleep became natural, the body grew warmer each day, the appetite improved, the headaches were less frequent and severe, the bowels acted more naturally, and she so rapidly gained in physical strength that in less than three weeks she could walk a mile without great fatigue.

She was treated for six months, and recovered a large proportion of her original strength, and we believe that if we could have used oxygen inhalations during the same period she would have recovered it all. During one of her periods, and during treatment, she was attacked by serious flooding from the uterus. The hot water bag, filled with water at 115 Fahr., was immediately placed over the dorsolumbar sympathetic ganglia, with a resulting speedy relief. From the evidence set forth we see that oxygen, iron and the spinal ice bag will, each of them, relieve and cure the anemic condition. But is it not also evident that their combination will insure more speedy and much greater success ?

202 West 106th Street.

